# Retrospective Observational Study on Rebamipide Ophthalmic Suspension on Quality of Life of Dry Eye Disease Patients

**DOI:** 10.1155/2019/8145731

**Published:** 2019-05-02

**Authors:** Yuri Sakane, Masahiko Yamaguchi, Atsushi Shiraishi

**Affiliations:** ^1^Department of Ophthalmology, Ehime University Graduate School of Medicine, Shitsukawa, Toon, Ehime 791-0295, Japan; ^2^Ehime Prefectural Central Hospital, Matsuyama, Ehime 790-0024, Japan

## Abstract

**Purpose:**

The Dry Eye-Related Quality-of-Life Score (DEQS) is a Japanese dry eye-specific questionnaire that has been used to assess the symptoms of dry eye and their effects on the quality of life (QOL) in Japanese individuals. The purpose of this study was to determine the effect of rebamipide (RBM) on the QOL of Japanese patients with dry eye disease (DED).

**Method:**

The medical records of 43 patients (3 men and 40 women; mean age: 64 ± 14 years; range: 32 to 83 years), who were diagnosed with DED and treated with RBM at the Ehime University Hospital between November 2012 and June 2016, were reviewed. The effects of 2% rebamipide (RBM) ophthalmic suspension on the symptoms of DED was determined by the answers to the DEQS questionnaire and clinical findings. The clinical findings before and 1, 3, 6, 12, and 24 months after initiating the RBM treatment were reviewed. The following data were collected from the DEQS: the Summary score and two subscale scores, the Bothersome ocular symptoms score, and the Impact on daily life score. In addition, the standard fluorescein staining score, the Schirmer I test score, and the tear breakup time (TBUT) score were analyzed.

**Result:**

The Summary score and Impact of daily life score of the DEQS were improved significantly after 1, 3, 6, and 12 months of RBM, and the Bothersome ocular symptoms scores of the DEQS were also improved after 1, 3, and 6 months. The fluorescein staining scores were significantly decreased after 1, 3, 6, and 12 months, and the TBUT score was significantly increased after 1 month.

**Conclusion:**

RBM treatment improves the QOL by alleviating the corneal and conjunctival epithelial damages. The DEQS is a useful questionnaire that can assess the severity of the DED symptoms and their impact on the QOL. This trial is registered with UMIN000024405.

## 1. Introduction

Dry eye disease (DED) is a multifactorial ocular surface disorder that is characterized by a breakdown of the homeostasis of the tear film causing various symptoms and visual disturbances. The report of the National Eye Institute/Industry Workshop on Clinical Trials in Dry Eyes in 1995 emphasized the requirement of the presence of symptoms in the definition of DED [1], and this requirement was included in the Japanese definition of DED in 2006 [2]. The Bothersome symptoms and visual disturbances of DED have a negative impact on the daily activities such as reading, computer use, driving, and watching television [3]. The goals of treating DED are to improve the patient's ocular comfort and restoring the ocular surface and tear film to the normal homeostatic state [4]. Therefore, a comprehensive questionnaire that assesses the symptoms and the impact of DED on the quality of life (QOL) is as important as the clinical findings.

The Dry Eye-Related Quality-of-Life Score (DEQS) is a Japanese dry eye-specific questionnaire that can be used to assess the symptoms of dry eye and their effects on the QOL [5]. The DEQS has good reliability, validity, specificity, and responsiveness, and it has been shown to be helpful in assessing the QOL in several clinical studies [6–9]. Because the DEQS is the only dry eye-specific questionnaire that has been validated in Japanese individuals, its use is expected to increase in future clinical studies on DED in Japan. The DEQS is appropriate for evaluating the changes in the DED conditions and the therapeutic effects. Utsunomiya et al. [7] reported the cutoff score for DED diagnosis by the DEQS may be set at 15.

Rebamipide (RBM) ophthalmic suspension (Mucosta Ophthalmic Suspension UD2%; Otsuka Pharmaceutical Co. Ltd., Tokyo, Japan) has recently become commercially available as a mucin secretagogue in Japan. RBM ophthalmic suspension has been shown to increase the production of mucins from the cornea and conjunctiva, and it has improved the objective findings of the ocular surface and subjective symptoms in patients with DED [10]. In addition, RBM has been shown to have anti-inflammatory effects [11, 12]. Kinoshita et al. reported the long-term effect of RBM on the objective signs and subjective symptoms [13]. They reported that the scores of the subjective symptoms were significantly improved at week 2 compared with those at the baseline, and further improvements of the scores were observed at every visit up to week 52. However, there have not been any studies that reported the long-term effect of RBM on the QOL of patients with DED obtained by a validated questionnaire.

Thus, the purpose of this study was to determine the long-term effects of RBM on the symptoms of DED and the QOL. To accomplish this, patients with DED were requested to answer the DEQS questionnaire before and during the treatment with RBM.

## 2. Methods

This was a retrospective case series study. The procedures used conformed to the tenets of the Declaration of Helsinki and were approved by the Internal Review Board of the Ehime University Hospital. The study was registered with the University Hospital Medical Information Network in Japan (number UMIN000024405). Informed consent was obtained in the form of opt-out on the website.

The diagnostic criteria used conformed to those defined by the Japanese Dry Eye Society in 2006 [2] and were used to diagnose DED based on the presence of 2 or more of the following 3 items: the presence of symptoms associated with dry eye including ocular discomfort such as dry sensation, irritation, foreign body sensation, pain, and other symptoms; an abnormality of the tear film breakup time (TBUT) of ≤5 seconds or a Schirmer I test value ≤ 5 mm; and the presence of keratoconjunctival epithelial disorders with a fluorescein staining score ≥3 and a maximum score of 9.

### 2.1. Subjects

Outpatients who were diagnosed with DED and treated with RBM in the Ehime University Hospital between April 2013 and June 2016 were initially screened for this study. The eligible subjects were ≥20 years and had been followed up for at least one month. The subjects were excluded if any of the following was found: active ocular infection, ocular inflammation, abnormal eyelids or blinking, or history of ocular surgery that could have an influence on the cornea and tears during the investigation period.

### 2.2. Assessments

We reviewed the clinical findings obtained before and 1, 3, 6, 12, and 24 months after initiating the RBM treatment. The following data were collected: the demographic characteristics, the DEQS results, fluorescein staining score, Schirmer I test, and tear breakup time (TBUT).

The DEQS consists of 15 items and 2 subscales including the “Bothersome effects of the ocular symptoms (6 items)” and the “Impact of dry eye on daily life (9 items)” scores. We evaluated three scores of the DEQS: the Summary score, the Bothersome ocular symptoms score, and the Impact on daily life score. All of the scores ranged from 0 to 100, with a higher score representing a greater disability.

The fluorescein ocular surface staining test was performed with observations through a blue-free barrier filter. It was used to determine whether the corneal and conjunctival epithelium was damaged. According to the van Bijsterveld system [14], the ocular surface was divided into three zones: the nasal bulbar conjunctiva, the cornea, and the temporal bulbar conjunctiva. The maximum staining score for each area was 3 points, and the maximum staining score for the overall surface was 9 points. The Schirmer I test was performed for 5 minutes without topical anesthesia. In most cases, the Schirmer I test was not measured after beginning the RBM treatment; therefore, only the results before the initial administration were recorded. The TBUT score was measured with observations of the cornea and conjunctiva through a slit lamp after an instillation of fluorescein into the conjunctival sac. The time from normal blinking to the first appearance of a dry spot in the tear film was measured three times and the average was used for the statistical analyses.

### 2.3. Statistical Analyses

Parametric or nonparametric tests were used for all analyses with descriptive statistics and statistical testing according to the results of the Kolmogorov–Smirnov tests. The values of the different parameters are presented as the means ± standard deviations. The eye with the higher fluorescein staining score was used for the statistical analyses, or if the scores were the same in the two eyes, the scores of the right eye were used. One sample paired *t* tests or Wilcoxon's signed-rank tests were used to determine the significance of the differences between two groups at the baseline and at each visit. Pearson's product-moment correlations or Spearman's rank-order correlation analyses were performed to determine the significance of the correlations between different parameters. All tests were 2-tailed, and *p* <0.05 was taken to be statistically significant. The statistical analyses were performed using the SAS software, ver.9.3.

## 3. Results

Forty-three patients with DED were studied. There were 3 men and 40 women with a mean age of 64 ± 14 years. The characteristics of participants before initiating the RBM treatment are shown in Table 1.

The number of subjects who completed the DEQS questionnaire was 25 at 1 month, 17 at 3 months, 19 at 6 months, 9 at 12 months, and 8 at 24 months. The number of subjects at each analysis period was different because the reexamination times differed; e.g., some patients visited at 1 month and 6 months and other patients visited at 3, 6, and 12 months after initiating the RBM treatment. Thus, patients who were not included in the 1-month period may be included in the 3-month period. Only 4 subjects completed all the visits, and it was too few for statistical analysis.

The Summary score and Impact on daily life score of the DEQS questionnaire indicated a significant improvement over the corresponding scores at the baseline and at 1, 3, 6, and 12 months. The Bothersome ocular symptoms score was significantly improved at 1, 3, and 6 months (Table 2).

The fluorescein staining score was obtained from 22 patients at 1 month, 14 at 3 months, 17 at 6 months, 9 at 12 months, and 7 at 24 months. The fluorescein staining score was significantly lower than the baseline scores at 1, 3, 6, and 12 months (Table 3). The TBUT score was also improved significantly at 1 month, but the sample size after 3 months were too few for statistical analyses (Table 3).

The correlations between the scores of DEQS and the clinical findings were analyzed in subjects where both sets of data were available. There were no significant correlations between the Summary score of DEQS, the fluorescein staining score, and the TBUT score at all time points (Table 4).

The most frequently observed adverse event during the RBM treatment was eye pain which was reported by 6 patients (14.0%). Foreign body sensations and eye discharge were observed in three patients (7.0%), dryness and redness were observed in two patients (4.7%), and an itching sensation and a bitter taste were observed in one patient (2.3%). However, no serious treatment-related adverse events occurred in any subject. Three patients were discontinued because of the development of adverse events (2 patients at 3 months and 1 patient at 6 months).

## 4. Discussion

Our results showed that the DEQSs were significantly improved as soon as one month after initiating the RBM treatment, and the improvements were reported throughout the treatment period. Similar improvements were found in the fluorescein staining scores and TBUT score after initiating the RBM treatment.

RBM was launched in the Japanese market in 2012 for the treatment of DED. Rebamipide is a mucoprotective drug, and it has been widely used for the treatment of gastric and duodenal ulcers. RBM has been shown to increase the production of mucins from corneal and conjunctival tissues [15, 16], increase the number of goblet cells [16], and increase the anti-inflammatory properties of the tissues [11, 12]. Because of these properties, RBM is considered to be an effective agent to improve the signs and symptoms of DED.

Kinoshita et al. performed a 52-week study of RBM in patients with DED and reported that all the objective signs, viz., fluorescein corneal staining, lissamine green conjunctival staining, and TBUT, and the subjective symptoms scores were significantly improved at week 2 compared with that at the baseline [13]. They also reported that further improvements were observed at almost every visit up to 52 weeks. Another 12-week study that evaluated the effect of RBM treatment also reported that the dry eye symptoms scores and fluorescein ocular surface staining score were significantly improved at 2 weeks and maintained until 12 weeks [10]. In our study, the DEQS and the fluorescein staining score were improved at 1 month which indicated that the RBM treatment was effective from an early time. But, we could not compare among the later treatment periods. Unfortunately, we could only compare baseline values for each visit due to differences in the constitution of the subjects at each follow-up visit.

The strength of our study is the use of the DEQS to assess the subjective symptoms of DED and its impact on the QOL. Previous studies neither used validated questionnaire to assess the subjective symptoms nor evaluated the impact on the QOL. In this study, the Summary score and one of the subscale score of the DEQS which are associated with the activities of daily living were significantly improved at 1, 3, 6, and 12 months after the RBM treatment. The Bothersome ocular symptoms score was significantly improved at 1, 3, and 6 months after beginning the RBM treatment. These results indicated that RBM is effective in improving the symptoms of DED during the treatment periods, especially their impact on the QOL. On the contrary, the Summary score and both subscale scores showed no significant difference at 24 months after the RBM treatment compared to that before the RBM treatment. This may be caused by the decrease in the sample size due to the improvement of the DED and a discontinuation of the treatment or transfer to neighboring clinic. However, the scores were not significantly different between 12 and 24 months.

Utsunomiya et al. [7] reported that the cutoff score for DED diagnosis by the DEQS can be set at 15. In a validation study of DEQS [5], the average Summary score of DEQS was 33.7 in patients with DED and 6.0 in normal subjects. In this study, the average Summary scores of DEQS before the RBM treatment were high (Table 2). After initiating the RBM treatment, the average Summary scores were improved significantly, but it still remained higher than normal. The higher scores of the DEQS might be related to neuropathic pain which has received increasing recognition as a factor in DED [17, 18]. Neuropathic pain is defined as pain caused by a lesion or disease of the somatosensory nervous system. DED patients with neuropathic pain have more severe and chronic DED symptoms such as a spontaneous burning pain and pain evoked by wind and light. Nonresponses to therapies that target the ocular surface and tears and discordance between symptoms and clinical signs are also features suggestive of a neuropathic component to dry eye symptoms. In addition, the fluorescein staining scores were <3 points at all visit, and discrepancy between subjective symptoms and clinical findings was found. The inclusion of patients with neuropathic pain might have limited the improvement of the DEQS after the RBM treatment.

The fluorescein staining score was significantly improved at 1, 3, 6, and 12 months which is similar to the results of the DEQS. The TBUT score was significantly improved at 1 month; however, the number of patients after 3 months was too few for statistical analyses. Although the TBUT score was not found to be significantly improved, RBM improved the corneal and conjunctival epithelial damage as well as the symptoms of DED. The effect of these changes led to improvements of the QOL during the treatment period. Similarly, RBM has been shown to be effective for other ocular surface diseases such as allergic conjunctivitis, lid wiper epitheliopathy, and superior limbic keratoconjunctivitis [19–22].

Although the DEQSs and the fluorescein staining scores improved during the treatment periods, the correlations between the changes of the fluorescein staining scores and the DEQS scores were not significant at all time points. Similar disagreements between clinical findings and subjective symptoms of DED have been reported [23–25]. This discrepancy can be explained by the natural variations of the disease processes, the “subjective” nature of the symptoms, and the variability in pain thresholds, and cognitive responses to questions about physical sensations of the eyes [26]. On the contrary, DED patients generally seek medical treatment to alleviate the irritating ocular symptoms that affect their QOL, and they want an improvement of their QOL rather than an improvement of the clinical findings. Therefore, a validated questionnaire that assesses the symptoms, vision-related functions, and the impact of the DED on their QOL is needed for assessing the therapeutic effects of anti-DED agents. To evaluate the therapeutic effect properly, a simple, reproducible, reliable, and quantitative questionnaire is necessary.

Many of the instruments used for assessing the DED symptoms and their impact on the QOL are time consuming, and their ability to quantify changes is limited. In 2017, the Tear Film and Ocular Surface (TFOS) and Dry Eye Workshop II (DEWS II) reports summarized the twelve validated questionnaires for DED [27]. Among them, 5 questionnaires, viz., the National Eye Institute Visual Function Questionnaire-25 (VFQ-25), the Ocular Surface Disease Index (OSDI), the Impact of Dry Eye on Everyday Life (IDEEL), the North Carolina Dry Eye Management Scale (UNC DEMS), and the DEQS, included items related to the QOL. The VFQ-25 is probably the most widely used questionnaire that is used to assess the visual function and vision-related QOL [28–30]. However, the VFQ-25 is not disease specific; thus, it may not be suitable for evaluating more subtle changes of the symptoms. The OSDI and the IDEEL are frequently used to assess the severity of DED. The OSDI is very useful for diagnosing and evaluating the severity of the symptoms [31], but it has some limitations in that it does not fully cover the impact of DED on the QOL such as the psychological and social aspects. The IDEEL is a 57-item questionnaire that was developed to evaluate the QOL, dry eye symptoms, and treatment satisfaction [32]. The IDEEL includes all relevant domains of DED; however, it is not easy to use in routine clinical practice because of its long testing time of approximately 30 min. The UNC DEMS is a single-item questionnaire that asks DED patients to rate their symptoms and the effects of those symptoms on their daily life [33]. Because the UNC DEMS was created as a quick and reliable measure, it is not suitable for evaluating the DED symptoms in detail.

The DEQS questionnaire was developed to evaluate the symptoms of DED and the effects of DED on the activities of daily living. Our experience with its use on DED patients showed that it can be used easily in routine clinical practice. It is easy for patients to answer the DEQS questionnaire while waiting for their examination because the DEQS requires approximately 5 min for completion. The DEQS questionnaire was recently used to assess the QOL in dry eye patients and also to determine the effectiveness of different kinds of dry eye treatments [7–9, 34, 35]. Thus, we conclude that DEQS is an appropriate method to evaluate the therapeutic effects of different types of treatments for DED.

This retrospective study had limitations. Although the objective and subjective data were collected from each subject during the treatment period, the number of collected data decreased due to an improvement of the DED and a discontinuation of the treatment or transfer to a neighborhood clinic by the patients. Therefore, statistical comparisons became difficult. In particular, the TBUT score was not routinely recorded after beginning the RBM treatment. However, the DEQS questionnaire score and the fluorescein staining score were significantly improved. This suggests that RBM is effective for treating DED and can improve the clinical signs and the QOL. The answers to the DEQS questionnaire showed that there was a significant improvement up to 12 months indicating that the DEQS is appropriate for evaluating changes in the DED conditions and in determining the therapeutic effects of RBM [6–9].

In conclusion, the DEQS questionnaire is a useful tool that can be used in routine clinical practice to assess the symptoms of DED and their impact on the QOL. Topical RMB can improve the corneal and conjunctival epithelial damage, the symptoms of DED, and the QOL during the treatment period. The DEQS questionnaire can be used to evaluate the therapeutic effects of a treatment regimen and to follow the symptoms of DED patients.

## Figures and Tables

**Table 1:** Characteristics of participants before initiating the RBM treatment.

Parameters	
Age, mean ± SD (range), years	64 ± 14 (32–83)
Sex (male : female)	3 : 40
DEQS questionnaire	
Summary score, mean (SD), point	41.7 (22.7)
Bothersome ocular symptoms score, mean (SD), point	48.1 (23.1)
Impact on daily life score, mean (SD), point	37.7 (25.0)
Fluorescein staining score, mean (SD), point	2.3 (2.1)
Tear film breakup time score, mean (SD), s	1.9 (0.5)
Schirmer's testing, mean (SD), mm	8.9 (10.6)

**Table 2:** DEQS questionnaire score at each visit.

DEQS questionnaire score	1 month (*n*=25)			3 months (*n*=17)			6 months (*n*=19)			12 months (*n*=9)			24 months (*n*=8)		
	Before	1 month	*p* value	Before	3 months	*p* value	Before	6 months	*p* value	Before	12 months	*p* value	Before	24 months	*p* value
Summary score	51.4 (24.4)	37.7 (23.2)	**0.008**	53.9 (23.9)	42.9 (28.3)	**0.018**	57.5 (25.9)	41.5 (21.7)	**0.001**	49.5 (19.4)	39.4 (23.8)	**0.049**	41.1 (24.7)	42.2 (16.0)	0.58
Bothersome ocular symptoms score	38.2 (26.9)	31.9 (27.1)	**0.019**	42.0 (26.6)	37.7 (24.8)	**0.016**	43.3 (20.5)	29.8 (21.7)	**0.02**	46.0 (17.2)	29.3 (19.1)	0.180	39.7 (19.2)	29.2 (13.8)	0.82
Impact on daily life score	43.0 (23.6)	34.2 (24.6)	**0.014**	44.5 (24.1)	39.8 (24.8)	**0.004**	53.2 (20.0)	34.4 (20.8)	**0.001**	47.4 (16.3)	33.3 (17.1)	**0.034**	41.0 (20.3)	34.4 (12.1)	0.40

Values are the means (±SDs). One sample paired *t* test was used for statistical comparisons.

**Table 3:** Clinical findings at each visit.

	1 month			3 months			6 months			12 months			24 months		
	Before	1 month	*p* value	Before	3 months	*p* value	Before	6 months	*p* value	Before	12 months	*p* value	Before	24 months	*p* value
	*n*=22			*n*=14			*n*=17			*n*=9			*n*=7		
Fluorescein staining score	1.8 (2.1)	1.2 (1.6)	**0.008**	2.4 (2.5)	1.4 (1.3)	**0.003**	2.8 (2.5)	1.7 (1.7)	**0.011**	2.3 (2.1)	0.9 (1.1)	**0.049**	1.9 (1.7)	0.9 (1.4)	0.17
	*n*=6			*n*=3			*n*=4			*n*=2			*n*=1		
Tear breakup time (TBUT, s) score	2.1 (0.5)	4.1 (1.3)	**0.042**	2.3 (0.3)	3.3 (0.3)	0.25	2.3 (0.3)	3.3 (2.0)	0.50	1.8 (0.3)	1.5 (0.7)	1.0	1.0	2.0	

Values are the means (±SD). Wilcoxon's signed-rank test was used for statistical comparisons.

**Table 4:** The correlations between the DEQS and the clinical findings.

	Summary score of DEQS											
	Before		1 month		3 months		6 months		12 months		24 months	
	*r*	*p* value	*r*	*p* value	*R*	*p* value	*r*	*p* value	*r*	*p* value	*r*	*p* value
	*n*=43		*n*=22		*n*=14		*n*=17		*n*=9		*n*=7	
Fluorescein staining score	−0.01	0.97	0.29	0.19	0.08	0.78	−0.31	0.23	−0.12	0.76	0.26	0.58
	*n*=17		*n*=6		*n*=3		*n*=4		*n*=2		*n*=1	
Tear breakup time (TBUT, s) score	0.1	0.7	0.37	0.47	−1.0	0.44	0.6	0.4	−1		N/A	

*r* = correlation coefficient. N/A = not available. Spearman's rank-order correlation analyses were used for statistical comparisons.

## Data Availability

The data used to support the findings of this study are restricted by the Certified Review Board of Ehime University in order to protect patient privacy. The data are however available from the authors upon reasonable request and with permission of the Certified Review Board of Ehime University (rinri@m.ehime-u.ac.jp).
