# Corrigendum to “Retrospective Observational Study on Rebamipide Ophthalmic Suspension on Quality of Life of Dry Eye Disease Patients”

**DOI:** 10.1155/2020/8486704

**Published:** 2020-09-18

**Authors:** Yuri Sakane, Masahiko Yamaguchi, Atsushi Shiraishi

**Affiliations:** ^1^Department of Ophthalmology, Ehime University Graduate School of Medicine, Shitsukawa, Toon, Ehime 791-0295, Japan; ^2^Ehime Prefectural Central Hospital, Matsuyama, Ehime 790-0024, Japan

In the article titled “Retrospective Observational Study on Rebamipide Ophthalmic Suspension on Quality of Life of Dry Eye Disease Patients,” [1] there was an error in Table 2. The mean values were incorrectly entered for the summary score, the bothersome ocular symptoms score, and the impact on daily life score. The authors apologize for this error and confirm that it does not affect the results and the conclusions of the article. The corrected table is as follows.

## Figures and Tables

**Table 2:** DEQS questionnaire score at each visit.

DEQS questionnaire score	1 month, *n* = 25			3 months, *n* = 17			6 months, *n* = 19			12 months, *n* = 9			24 months, *n* = 8		
	Before	1M	*p* value	Before	3M	*p* value	Before	6M	*p* value	Before	12M	*p* value	Before	24M	*p* value
Summary score	43.0 (23.6)	34.2 (24.6)	0.008	44.5 (24.1)	39.8 (24.8)	0.018	53.2 (20.0)	34.4 (20.8)	0.001	47.4 (16.3)	33.3 (17.1)	0.049	41.0 (20.3)	34.4 (12.1)	0.58
Bothersome ocular symptoms score	51.4 (24.4)	37.7 (23.2)	**0.019**	53.9 (23.9)	42.9 (28.3)	**0.016**	57.5 (25.9)	41.5 (21.7)	**0.02**	49.5 (19.4)	39.4 (23.8)	0.180	41.1 (24.7)	42.2 (16.0)	0.82
Impact on daily life score	38.2 (26.9)	31.9 (27.1)	**0.014**	42.0 (26.6)	37.7 (24.8)	**0.004**	43.3 (20.5)	29.8 (21.7)	**0.001**	46.0 (17.2)	29.3 (19.1)	**0.034**	39.7 (19.2)	29.2 (13.8)	0.40

Values are the means (±SDs). One sample paired *t*-test was used for statistical comparisons.

## References

[1] Sakane Y., Yamaguchi M., Shiraishi A. (2019). Retrospective observational study on Rebamipide ophthalmic suspension on quality of life of dry eye disease patients. *Journal of Ophthalmology*.

